# Traumatic Pulmonary Embolism

**DOI:** 10.7759/cureus.64614

**Published:** 2024-07-15

**Authors:** Karthik Ventrapragada, Alexis Wright, Sana Tahir, Lisa Tartaglia, Latha Ganti

**Affiliations:** 1 Biomedical Sciences, University of Central Florida, Orlando, USA; 2 Medical School, Lewis Katz School of Medicine, Temple University, Philadelphia, USA; 3 Medicine, Orlando College of Osteopathic Medicine, Winter Garden, USA; 4 Specialty Care, Orlando College of Osteopathic Medicine, Winter Garden, USA; 5 Emergency Medicine and Neurology, University of Central Florida, Orlando, USA; 6 Research, Orlando College of Osteopathic Medicine, Winter Garden, USA; 7 Medical Sciences, The Warren Alpert Medical School of Brown University, Providence, USA

**Keywords:** ground-level fall, trauma, pulmonary embolism, rib fracture, traumatic pulmonary embolism

## Abstract

Pulmonary embolism (PE) is a life-threatening illness caused by a blood clot obstructing a pulmonary artery, resulting in decreased blood supply to the lungs. PE is a high-stakes diagnosis with considerable morbidity and death if left untreated. This case emphasizes the increased risk of PE associated with trauma and stresses the importance of this differential diagnosis in patients who report dyspnea following physical trauma. Understanding the risk factors and processes that contribute to PE in trauma patients is critical for accurate diagnosis and treatment.

## Introduction

Traumatic pulmonary embolism (TPE) is a significant but often under-recognized complication following trauma, especially in patients with major orthopedic injuries, traumatic brain injuries, or spinal cord injuries. The condition contributes to morbidity and mortality in trauma populations and requires heightened clinical awareness for timely diagnosis and management [1-3]. Patients with acute pulmonary embolism (PE) have significant variability in short-term mortality, from less than 0.6% in low-risk patients to 19% in high-risk patients [4].

The incidence of PE following trauma varies widely in the literature, but it is generally acknowledged that trauma patients are at an increased risk due to hypercoagulability induced by tissue injury and immobility. A 2020 review reports that the incidence of PE among trauma patients varies considerably, ranging from 0.35% to 24%. The incidence of early post-traumatic PE varies widely, from 10% to 42% [3].

The pathophysiology of PE involves a triad of risk factors known as Virchow's triad: venous stasis, endothelial injury, and hypercoagulability. Trauma particularly enhances these mechanisms. Endothelial damage and blood stasis result directly from physical injury, while hypercoagulability results from the systemic inflammatory response to trauma [5,6]. Patients with severe traumatic injuries such as spinal cord injuries, traumatic brain injuries, lower extremity fractures, and severe chest trauma are at high risk for hypercoagulability and thromboembolism, or TPE [7].

Diagnosing TPE involves a combination of clinical judgment, imaging studies, and biomarkers. The gold standard for diagnosing PE is computed tomography pulmonary angiography (CTPA), which provides direct visualization of clots within the pulmonary arteries. As a stand-alone test, it is nonspecific. A study of 19,258 patients over a six-year period demonstrated that D-dimer was elevated in various pathologies, including aortic aneurysm, respiratory infection, leukemia, and stroke [8]. Although nonspecific, D-dimer levels can help guide the need for further imaging in patients who are at low or moderate risk [4].

## Case presentation

A 51-year-old man presented to the emergency department (ED) a week after he sustained a ground-level fall onto his left side. He assumed he had bruised some ribs and tried to manage his pain at home with acetaminophen, gabapentin, and methocarbamol. He had these medications at home due to his history of chronic back pain. The pain became worse rather than better over a week, and he also developed shortness of breath. His wife became concerned and insisted he go to the ED. Upon ED arrival, his vital signs were as follows: temperature 98.2 °F, blood pressure 142/75 mmHg, pulse 66 beats per minute, respiratory rate 18 breaths per minute, and oxygen saturation 98% on room air.

**Figure 1:**
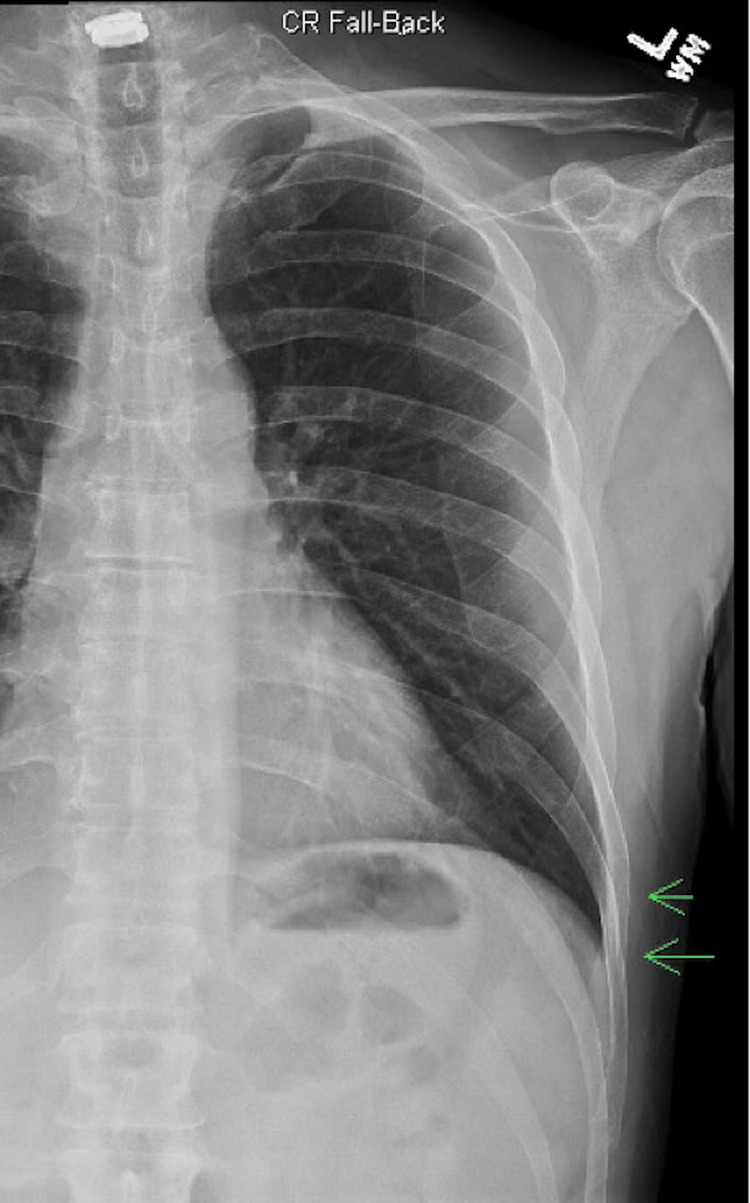
Chest radiograph demonstrating two rib fractures on the left (arrows)

The physical examination revealed a slender man who appeared uncomfortable but was in no respiratory distress. Palpation of the left lower ribs revealed point tenderness. The remainder of the physical exam was unremarkable, including the presence of normal bilateral breath sounds. He was given 30 mg of intravenous ketorolac and 4 mg of morphine for analgesia. Chest radiographs revealed left lateral eighth and ninth rib fractures (Figure 1).

**Figure 2:**
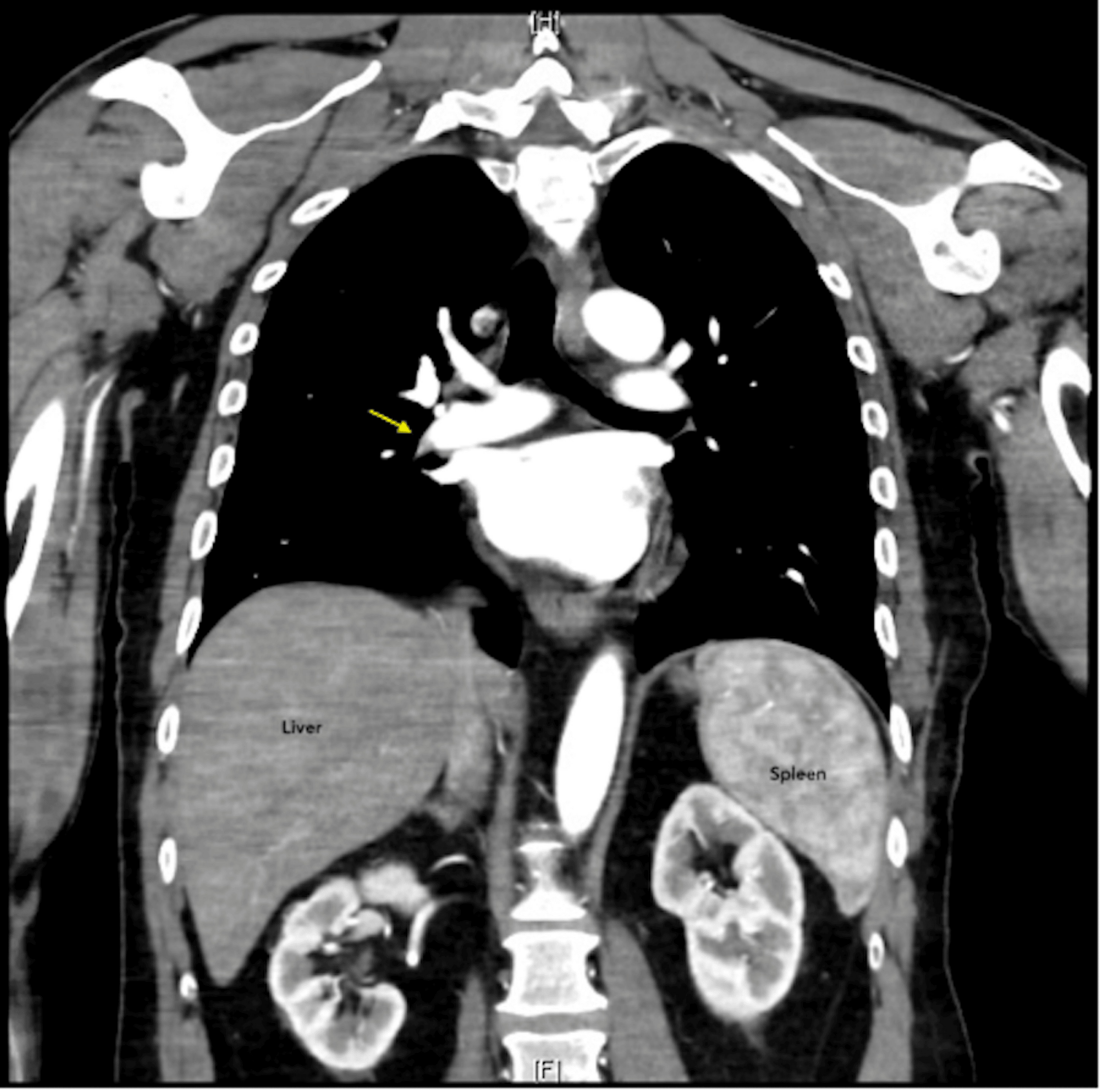
Chest tomography angiogram (CTA) demonstrating a filling defect consistent with a pulmonary embolism (arrow)

Due to his dyspnea, a chest CT angiography was performed, which detected a PE (Figure 2).

The patient was admitted to the hospital to initiate anticoagulant therapy and pain management. He had an uneventful discharge on day four. The laboratory analyses are summarized in Table 1.

**Table 1:** The patient's chemistry panel BUN: blood urea nitrogen

Lab	Reference Range	Patient Value
Sodium	135–145 mmol/L	139
Potassium	3.5–5.3 mmol/L	3.5
Chloride	98–107 mmol/L	104
Carbon dioxide	21–32 mmol/L	26
Anion gap	4–12 mmol/L	9
BUN	7–18 mg/dL	15
Creatinine	0.6–1.3 mg/dL	1.1
Estimated glomerular filtration rate	> 60	> 60
Glucose	74–106 mg/dL	111 H
Calcium	8.4–10.2 mg/dL	9.0
Total bilirubin	0.0–1.0 mg/dL	0.5
Aspartate aminotransferase	15–37 U/L	30
Alanine aminotransferase	4–36 U/L	28

## Discussion

The presented case showcases the significance of TPEs as a potential complication following blunt thoracic trauma, particularly in the context of rib fractures. Rib fractures are associated with complications such as pneumonia and respiratory compromise; however, the development of TPE adds an additional layer of complexity to the management of such patients [9].

TPEs result from the dislodgment of thrombi formed in the deep veins of the lower extremities or pelvis, leading to their migration to the pulmonary vasculature [10]. Blunt thoracic trauma, as seen in this case study of a ground-level fall resulting in rib fractures, can predispose individuals to the development of TPE through mechanisms such as venous stasis, endothelial injury, and activation of the coagulation cascade [10].

In the presented case, the patient's delayed presentation to the ED following the fall allowed for the progression of symptoms, including shortness of breath. While the initial focus was on managing pain, it is crucial to recognize the potential for TPE in patients with blunt thoracic trauma, particularly those with associated risk factors such as immobilization or underlying hypercoagulable states [11].

The clinical presentation of TPE can vary widely, ranging from asymptomatic incidental findings on imaging to life-threatening respiratory compromise and hemodynamic instability. In this case, the absence of overt signs of respiratory distress upon ED arrival highlights the challenges associated with diagnosing TPE based solely on clinical examination. However, the development of shortness of breath in the context of rib fractures should prompt consideration of TPE as a potential complication warranting further evaluation and diagnostic imaging. CTPA is the imaging modality of choice for diagnosing TPE, as it offers high sensitivity and specificity for detecting PE. Other forms of testing include troponin levels, brain natriuretic peptide, and ECG [12].

The management of TPE involves anticoagulation therapy to prevent further thrombus propagation and embolic events [13]. However, the initiation of anticoagulation in patients with traumatic injuries, such as rib fractures, requires careful consideration of the risk of hemorrhage versus the benefit of preventing TPE-related morbidity and mortality. Individualized decision-making based on the patient's clinical presentation, comorbidities, and risk factors is essential in guiding the choice and timing of anticoagulation therapy in these cases.

The presented case highlights the importance of considering TPEs as a potential complication following blunt thoracic trauma, particularly in patients with associated risk factors such as rib fractures. Early recognition, prompt diagnosis, and appropriate management are essential in mitigating the morbidity and mortality associated with TPE in this population.

## Conclusions

This case report highlights the importance of recognizing the potential for PE in patients with a history of traumatic injury. The patient's fall and subsequent rib fractures served as precipitating factors for the development of TPE. The timely utilization of diagnostic tools, such as chest CT angiography, allowed for accurate identification of the embolism. Prompt initiation of anticoagulant therapy and pain management contributed to a successful treatment outcome and uneventful hospital discharge. This case emphasizes the need for clinicians to remain vigilant and consider PE as a potential complication in trauma patients.
